# Supplementary material for: Molecular Network and Culture Media Variation Reveal a Complex Metabolic Profile in Pantoea cf. eucrina D2 Associated with an Acidified Marine Sponge
Source: Int J Mol Sci. 2020 Aug 31;21(17):6307. doi: 10.3390/ijms21176307 (PMC7504411; doi:10.3390/ijms21176307)
Supplement: Supplementary file 1 [file ijms-21-06307-s001.pdf]

# Molecular Network and Culture Media Variation Reveal a Complex Metabolic Profile in *Pantoea cf. eucrina* D2 Associated to an Acidified Marine Sponge

**Giovanni Andrea Vitale[1], Martina Sciarretta[2], Chiara Cassiano[2], Carmine Buonocore[1], Carmen Festa[2], Valerio Mazzella[3], Laura Núñez Pons[3], Maria Valeria D'Auria[2*], Donatella de Pascale[1,4*]**

[1] Institute of Biochemistry and Cell Biology, National Research Council, 80131 Naples, Italy; giovanniandrea.vitale@ibbc.cnr.it (G.A.V.), carmine.buonocore@ibbc.cnr.it (C.B.)

[2] Department of Pharmacy, University of Naples "Federico II", 80131 Naples, Italy; martina.sciarretta@unina.it (M.S.), chiara.cassiano@unina.it (C.C.), carmen.festa@unina.it (C.F.), madauria@unina.it (M.V.D.)

[3] Department of Integrated Marine Ecology (EMI), Stazione Zoologica Anton Dohrn, Villa Comunale, 80125 Naples, Italy; valerio.mazzella@szn.it (V.M), laura.nunezpons@szn.it (L.N.P)

[4] Department of Marine Biotechnology, Stazione Zoologica Anton Dohrn, Villa Comunale, 80125 Naples, Italy; donatella.depascale@szn.it (D.d.P.)

**Contents** S1

**Figure S1.** LC–ESI/HRMS analysis S2
**Figure S2.** Extracted Ion Chromatogram (XIC) S3
**Table S1.** Surfactins derivatives detected S4
**Table S2.** Lipoamino acids putative derivatives clustered in GNPS S5
**Table S3**: PCR protocol S8
**Table S4.** Mzmine parameters adopted to process MS data S8
**Table S5.** $^1$H and $^{13}$C NMR data of new compound **1** S9
**Table S6.** $^1$H and $^{13}$C NMR data of compounds **3**, **4** and **5** S10
**Figure S3.** $^1$H NMR spectrum of compound **1** S11
**Figure S4.** COSY spectrum of compound **1** S11
**Figure S5.** HSQC spectrum of compound **1** S12
**Figure S6.** HMBC spectrum of compound **1** S12
**Figure S7.** $^1$H NMR spectrum of compound **2** S13
**Figure S8.** $^1$H NMR spectrum of compound **3** S13
**Figure S9.** $^{13}$C NMR spectrum of compound **3** S14
**Figure S10.** $^1$H NMR spectrum of compound **4** S14
**Figure S11.** $^{13}$C NMR spectrum of compound **4** S15
**Figure S12.** $^1$H NMR spectrum of compound **5** S15
**Figure S13.** $^{13}$C NMR spectrum of compound **5** S16
**Figure S14.** $^1$H NMR spectrum of compound **6** S16
**Figure S15.** $^1$H NMR spectrum of compound **7** S17
**Figure S16.** $^1$H NMR spectrum of compound **8** S17
**Figure S17.** $^{13}$C NMR spectrum of compound **8** S18
**Figure S18.** Advanced Marfey's analysis of compound **3** S18
**Bibliography** S19

**Figure S1.** LC–ESI/HRMS analysis of *Pantoea eucrina* D2. (A) TIC (Mass Range 1000-1200) of the surfactin analogues in positive ion mode from the sample obtained in MSM C media. (B) Related Full-MS Spectrum.

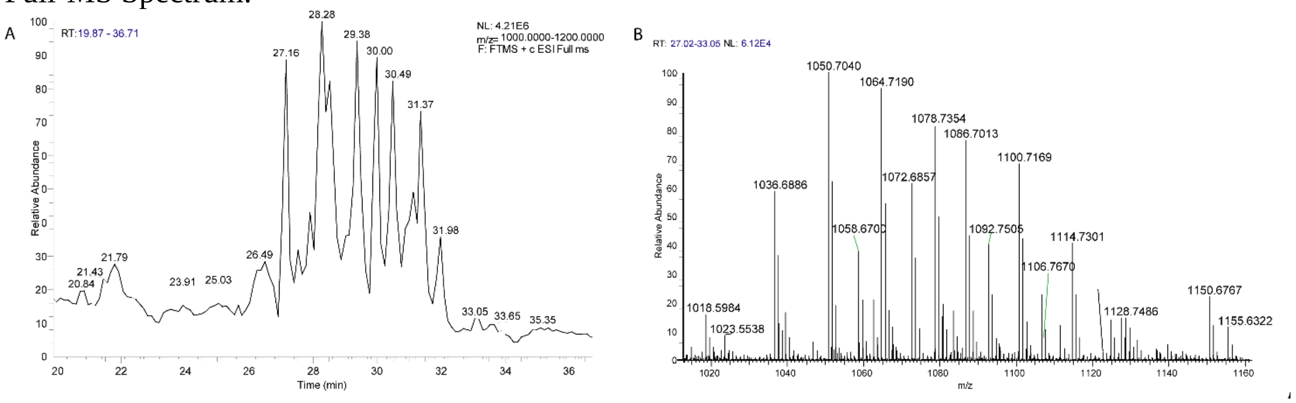

**Figure S2.** XICs of 1036.7, 1050.7, 1064.6, 1078.7, 1092.7 and 1106.7 *m/z* obtained from *Pantoea eucrina* D2 extracts.

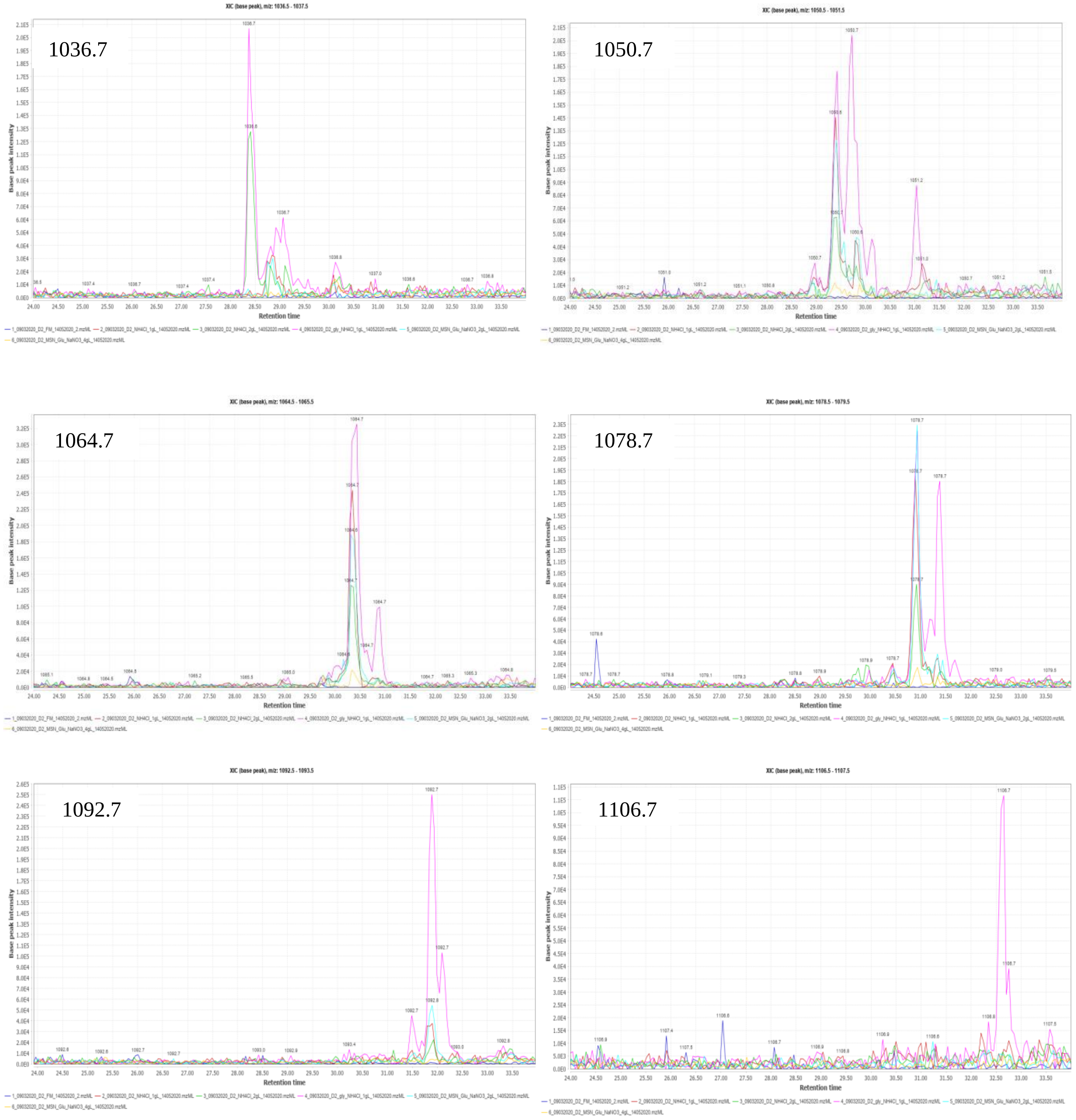

**Table S1.** Surfactins derivatives produced by *Pantoea eucrina* D2

| RT (min) | Mass (m/z) | Cyclopeptide sequence | β-OH-FA | Ref. |
|---|---|---|---|---|
| 27.1 | 1036.7 | β-OH-FA-E-L/I-L/I-V-L/I-D-L/I | C15 | [28] |
| 27.6 | 1036.7 | β-OH-FA-E-V-L/I-L/I-V-AME-V | C16 | new |
| 28.2 | 1050.7 | β-OH-FA-E-L/I-L/I-L/I-L/I-D-L/I | C15 | [28] |
| 28.8 | 1050.7 | β-OH-FA-E-L/I-L/I-V-L/I-D-L/I | C16 | [28] |
| 29.4 | 1064.7 | β-OH-FA-E-L/I-L/I-L/I-L/I-D-L/I | C16 | [28] |
| 30.0 | 1078.7 | β-OH-FA-E-L/I-L/I-L/I-L/I-D-L/I | C17 | [28] |
| 30.7 | 1092.7 | β-OH-FA-E-L/I-L/I-L/I-L/I-D-L/I | C18 | new |
| 31.2 | 1092.7 | β-OH-FA-E-L/I-L/I-L/I-AME-L/I-L/I | C17 | [29] |
| 32.0 | 1106.7 | β-OH-FA-E-L/I-L/I-L/I-AME-L/I-L/I | C18 | [30] |

**Table S2.** Lipoamino acids putative derivatives clustered in GNPS and manually dereplicated.

| N° | Adduct | Cluster index | Molecular formula | Precursor mass (Da) | RT consensus (min) | Typical fragment | Putative ID[a] | Error (ppm) |
|---|---|---|---|---|---|---|---|---|
| 1 | $[M+H]^+$ | 698 | $C_{26}H_{41}NO_3$ | 416.3149 | 27.6486 | Phe methyl ester typical fragment: 180 | PheME-C16:1 | -3.45 |
| 2 | $[M+H]^+$ | 677 | $C_{27}H_{43}NO_4$ | 446.3254 | 25.0672 | Phe typical fragment: 166 | Phe-C18:1-OH | -3.66 |
| 3 | $[M+H]^+$ | 713 | $C_{21}H_{41}NO_3$ | 356.3152 | 27.2696 | Leu methyl ester typical fragment: 146 | LeuMEC14 | -2.3 |
| 4 | $[M+H]^+$ | 924 | $C_{24}H_{39}NO_4$ | 406.2948 | 23.3338 | Phe typical fragment: 166 | Phe-C15-OH | -2.3 |
| 5 | $[M+H]^+$ | 845 | $C_{16}H_{31}NO_3$ | 286.2371 | 20.1012 | Leu typical frament: 132 | Leu-C10 | -3.78 |
| 6 | $[M+H]^+$ | 724 | $C_{28}H_{45}NO_4$ | 460.3409 | 27.0122 | Phe methyl ester typical fragment: 180 | PheME-C18:1-OH | -3.88 |
| 7 | $[M+H]^+$ | 772 | $C_{20}H_{37}NO_4$ | 356.2787 | 20.6244 | Leu typical frament: 132 | Leu-C15 | -2.58 |
| 8 | $[M+H]^+$ | 874 | $C_{25}H_{39}NO_3$ | 402.2993 | 24.5374 | Phe typical fragment: 166 | Phe-C16:1 | -3.78 |
| 9 | $[M+H]^+$ | 735 | $C_{24}H_{39}NO_4$ | 406.2941 | 24.2689 | Phe methyl ester typical fragment: 180 | PheME-C14-OH | -2.3 |
| 10 | $[M+H]^+$ | 712 | $C_{24}H_{43}NO_3$ | 394.3311 | 24.7277 | Leu typical fragment: 132 | Leu-C18:2 | -2.58 |
| 11 | $[M+H]^+$ | 819 | $C_{26}H_{41}NO_4$ | 432.3099 | 25.0434 | Phe methyl ester typical fragment: 180 | PheME-C16:1-OH | -3.43 |
| 12 | $[M+H]^+$ | 990 | $C_{27}H_{40}NO_3$ | 426.2969 | 27.2977 | Leu methyl ester typical fragment: 146 | LeuME-C19 | 9.19 |
| 13 | $[M+H]^+$ | 667 | $C_{20}H_{39}NO_4$ | 358.2945 | 21.843 | Leu typical fragment: 132 | Leu-C14-OH | -3.44 |
| 14 | $[M+H]^+$ | 719 | $C_{23}H_{37}NO_3$ | 376.2839 | 24.9617 | Phe typical fragment: 166 | Phe-C14 | -3.37 |
| 15 | $[M+H]^+$ | 914 | $C_{24}H_{45}NO_3$ | 396.3464 | 29.0843 | Leu methyl ester typical fragment: 146 | LeuME-C17:1 | -3.45 |
| 16 | $[M+H]^+$ | 680 | $C_{20}H_{39}NO_3$ | 342.2995 | 24.8123 | Leu typical fragment: 132 | Leu-C14 | -3.85 |
| 17 | $[M+H]^+$ | 679 | $C_{23}H_{45}NO_3$ | 384.3464 | 29.3992 | Leu methyl ester typical fragment: 146 | LeuME-C15 | -3.56 |
| 18 | $[M+H]^+$ | 681 | $C_{25}H_{41}NO_3$ | 404.3149 | 27.2696 | Phe typical fragment: 166 | Phe-C16 | -3.88 |
| 19 | $[M+H]^+$ | 886 | $C_{30}H_{49}NO_3$ | 472.3772 | 30.1405 | Phe typical fragment: 166 | Phe-C21:1 | -3.96 |
| 20 | $[M+H]^+$ | 782 | $C_{19}H_{37}NO_3$ | 328.2841 | 24.9171 | Leu methyl ester typical fragment: 146 | LeuME-C12 | -3.26 |
| 21 | $[M+H]^+$ | 939 | $C_{23}H_{35}NO_4$ | 390.2626 | 21.039 | Phe typical fragment: 166 | Phe-C14:1-OH | -4.7 |
| 22 | $[M+H]^+$ | 897 | $C_{26}H_{43}NO_3$ | 418.3307 | 28.3003 | Phe typical fragment: 166 | Phe-C17 | -3.39 |
| 23 | $[M+H]^+$ | 1255 | $C_{21}H_{41}NO_4$ | 372.3101 | 23.2758 | Leu typical fragment: 132 | LeuC15-OH | -3.45 |
| 24 | $[M+H]^+$ | 665 | $C_{22}H_{41}NO_3$ | 368.315 | 25.385 | Leu typical fragment: 132 | Leu-C16:1 | -3.99 |

| | | | | | | | | |
|---|---|---|---|---|---|---|---|---|
| 25 | $[M+H]^+$ | 860 | $C_{21}H_{33}NO_3$ | 348.2527 | 22.6887 | Phe typical fragment: 166 | Phe-C12 | -3.36 |
| 26 | $[M+H]^+$ | 690 | $C_{21}H_{41}NO_4$ | 372.3101 | 24.0387 | Leu methyl ester typical fragment: 146 | LeuME-C14-OH | -3.45 |
| 27 | $[M+H]^+$ | 1039 | $C_{22}H_{43}NO_4$ | 386.3257 | 25.5681 | Leu methyl ester typical fragment: 146 | LeuME-C15-OH | -3.45 |
| 28 | $[M+H]^+$ | 692 | $C_{25}H_{47}NO_4$ | 426.3567 | 26.9479 | Leu methyl ester typical fragment: 146 | LeuME-C18:1-OH | -3.83 |
| 29 | $[M+H]^+$ | 752 | $C_{23}H_{45}NO_4$ | 400.3411 | 26.3916 | Leu methyl ester typical fragment: 146 | LeuME-C16-OH | -3.96 |
| 30 | $[M+H]^+$ | 673 | $C_{25}H_{39}NO_3$ | 402.2994 | 25.5074 | Phe typical fragment: 166 | Phe-C16:1 | -3.53 |
| 31 | $[M+H]^+$ | 1146 | $C_{21}H_{31}NO_3$ | 346.2369 | 19.9967 | Phe typical fragment: 166 | Phe-C12:1 | -3.81 |
| 32 | $[M+H]^+$ | 943 | $C_{19}H_{37}NO_4$ | 344.2789 | 21.6497 | Leu methyl ester typical fragment: 146 | LeuME-C12-OH | -3.44 |
| 33 | $[M+H]^+$ | 664 | $C_{24}H_{45}NO_3$ | 396.3463 | 27.5224 | Leu typical fragment: 132 | Leu-C18:1 | -3.71 |
| 34 | $[M+H]^+$ | 686 | $C_{28}H_{45}NO_3$ | 444.3462 | 29.5672 | Phe methyl ester typical fragment: 180 | PheME-C18:1 | -3.53 |
| 35 | $[M+H]^+$ | 670 | $C_{24}H_{45}NO_4$ | 412.3411 | 24.749 | Leu typical fragment: 132 | LeuC18:1-OH | -3.84 |
| 36 | $[M+H]^+$ | 1302 | $C_{20}H_{39}NO_3$ | 342.2996 | 25.3334 | Leu methyl ester typical fragment: 146 | LeuME-C13 | -3.56 |
| 37 | $[M+H]^+$ | 863 | $C_{21}H_{39}NO_3$ | 354.2996 | 24.6844 | Leu methyl ester typical fragment: 146 | LeuME-C14:1 | -3.44 |
| 38 | $[M+H]^+$ | 895 | $C_{20}H_{37}NO_3$ | 340.2841 | 22.6048 | Leu typical fragment: 132 | Leu-C14:1 | -3.14 |
| 39 | $[M+H]^+$ | 770 | $C_{25}H_{45}NO_3$ | 408.3462 | 26.9268 | Leu methyl ester typical fragment: 146 | LeuME-C18:2 | -3.84 |
| 40 | $[M+H]^+$ | 674 | $C_{23}H_{37}NO_4$ | 392.2787 | 22.2598 | Phe typical fragment: 166 | Phe-C14-OH | -3.53 |
| 41 | $[M+H]^+$ | 682 | $C_{22}H_{41}NO_4$ | 384.3101 | 22.645 | Leu typical frament: 132 | LeuC16:1-OH | -3.34 |
| 42 | $[M+H]^+$ | 705 | $C_{25}H_{39}NO_4$ | 418.2941 | 23.0249 | Phe typical fragment: 166 | Phe-C16:1-OH | -3.91 |
| 43 | $[M+H]^+$ | 672 | $C_{25}H_{47}NO_3$ | 410.3619 | 29.753 | Leu methyl ester typical fragment: 146 | LeuME-C18:1 | -3.7 |
| 44 | $[M+H]^+$ | 678 | $C_{23}H_{43}NO_3$ | 382.3308 | 27.7683 | Leu methyl ester typical fragment: 146 | LeuME-C16:1 | -3.45 |
| 45 | $[M+H]^+$ | 2217 | $C_{27}H_{43}NO_3$ | 430.3304 | 27.6074 | Phe typical fragment: 166 | Phe-C18:1 | -4 |
| 46 | $[M+H]^+$ | 666 | $C_{22}H_{43}NO_3$ | 370.3308 | 27.1415 | Leu typical fragment 132 | Leu-C16 | -3.56 |
| 47 | $[M+H]^+$ | 979 | $C_{32}H_{53}NO_3$ | 500.4081 | 31.7338 | No clear fragments | | -4.54 |
| 48 | $[M+H]^+$ | 918 | $C_{19}H_{37}NO_4$ | 344.2786 | 21.0187 | Val typical fragment: 118 | Val-C14-OH | -4.31 |
| 49 | $[M+H]^+$ | 783 | $C_{23}H_{43}NO_3$ | 382.3306 | 26.7409 | Val typical fragment: 118 | Val-C18:1 | -3.97 |
| 50 | $[M+H]^+$ | 828 | $C_{20}H_{35}NO_3$ | 338.2679 | 20.8548 | Leu typical frament: 132 | Leu-C14:2 | -4.79 |
| 51 | $[M+H]^+$ | 745 | $C_{27}H_{41}NO_3$ | 428.315 | 25.0672 | Phe typical fragment: 166 | Phe-C18 | -3.43 |

| | | | | | | | | |
|---|---|---|---|---|---|---|---|---|
| 52 | $[M+H]^+$ | 758 | $C_{18}H_{35}NO_4$ | 330.2631 | 19.5006 | Leu typical fragment: 132 | Leu-C12-OH | -4.04 |
| 53 | $[M+H]^+$ | 740 | $C_{23}H_{43}NO_4$ | 398.3259 | 24.8327 | Leu methyl ester typical fragment: 146 | LeuME-C16:1-OH | -2.85 |
| 54 | $[M+H]^+$ | 878 | $C_{26}H_{43}NO_4$ | 434.325 | 26.5622 | Phe methyl ester typical fragment: 180 | PheME-C16-OH | -4.68 |
| 55 | $[M+H]^+$ | 721 | $C_{23}H_{35}NO_3$ | 374.2681 | 22.2598 | Phe typical fragment: 166 | Phe-C14:1 | -3.79 |
| 56 | [M+H]+ | 809 | $C_{23}H_{43}NO_3$ | 382.3306 | 26.8837 | Leu typical frament: 132 | Leu-C17:1 | -3.97 |
| 57 | [M+H]+ | 810 | $C_{22}H_{43}NO_3$ | 370.331 | 28.2473 | Leu methyl ester typical fragment: 146 | LeuME-C15 | -3.02 |

[a] Leu= leucine, Phe= pheylalanine, LeuME= leucine methyl ester, PheME= phenylalanine methyl ester

**Table S3**: PCR protocol

| | Step | Temperature | Time |
|---|---|---|---|
| | Initialization | 95°C | 5 min |
| 30 Cycles | Denaturation | 95°C | 30s |
| | Annealing | 55°C | 30s |
| | Elongation | 72°C | 2min |
| | Final extension | 72°C | 7min |
| | Hold | 4°C | ∞ |

**Table S4.** Mzmine parameters adopted to process MS data from *Pantoea eucrina* D2 extracts

| **Mass Detection** | | **Join aligner** | |
|---|---|---|---|
| MS1 Detection | 1·10^5 | m/z tolerance (ppm) | 20 |
| MS2 Detection | 1·10^2 | Weight for mass | 75 |
| **ADAP Chromatogram builder** | | RT tolerance (min) | 0.1 |
| Min group size in n° of scans | 4 | Weight for RT | 25 |
| Group intensity threshold | 1·10^5 | **Duplicate peak filter (NEW AVERAGE)** | |
| Min highest intensity | 1·10^5 | m/z tolerance (ppm) | 10 |
| m/z tolerance (ppm) | 20 | RT tolerance (min) | 0.05 |
| **Chromatogram deconvolution (Baseline cut-off)** | | **Peak filter** | |
| Min peak height | 1·10^5 | Keep only features with MSMS scan | V |
| Peak duration range (min) | 0-2 | **Gap Filling-Peak finder** | |
| Baseline level | 5·10^4 | Intensity tolerance | 10% |
| m/z range for MS2 scan pairing (Da) | 0.2 | m/z tolerance (ppm) | 20 |
| RT range for MS2 scan pairing (min) | 1.5 | RT tolerance (min) | 0.5 |

**Table S5.** $^1$H and $^{13}$C NMR data of new compound **1** (700 MHz and 175 MHz, $CDCl_3$)

| Position | $\delta_C$ type | $\delta_H$ m (*J* in Hz) | COSY | HMBC |
|---|---|---|---|---|
| NH | - | 5.97 m | H-2 | C1' small |
| 1 | 175.9 C | - | | - |
| 2 | 51.2 CH | 4.54 m | NH, H-3α, H-3β | C-1 |
| 3 | 40.6 $CH_2$ | 1.75[a] | H-2, H-3β | C1, C2, C4 |
| | | 1.62 m | H-3α, H-4 | |
| 4 | 25.0 CH | 1.73[a] | H-3β, $H_3$-5, $H_3$-6 | |
| 5 | 21.7 $CH_3$ | 0.96 d (5.9) | H-4 | C3, C4, C6 |
| 6 | 23.0 $CH_3$ | 0.97 d (5.9) | H-4 | C3, C4, C5 |
| 1' | 174.5 C | - | | - |
| 2' | 42.3 $CH_2$ | 2.50 d (14.7) | H2'α, H-3' | C1', C3', C4' |
| | | 2.34 dd (14.7, 8.9) | H-2'β, H-3' | |
| 3' | 68.9 CH | 4.00 m | $H_2$-2'α, $H_2$-3' | C1' |
| 4' | 36.9 $CH_2$ | 1.56 m | H-3', H-4'β; H-5'α | C3', C5' |
| | | 1.48 m | H3, H-4'α | |
| 5' | 25.4 $CH_2$ | 1.44 m | H-4'α | |
| | | 1.31[a] | | |
| 6' | 29.4 $CH_2$ | 1.30[a] | | |
| 7' | 29.4 $CH_2$ | 1.30[a] | | |
| 8' | 27.3 $CH_2$ | 2.00 m | H-7', H-9' | C7', C9', C10' |
| 9' | 130.1 CH | 5.36 m | H-8' | C8', C10' |
| 10' | 130.1 CH | 5.36 m | H-11' | C11' |
| 11' | 27.3 $CH_2$ | 2.00 m | H-10', H-12' | C9', C10', C12' |
| 12' | 29.4 $CH_2$ | 1.30[a] | | |
| 13' | 29.4 $CH_2$ | 1.30[a] | | |
| 14' | 29.4 $CH_2$ | 1.30[a] | | |
| 15' | 29.4 $CH_2$ | 1.30[a] | | |
| 16' | 31.9 $CH_2$ | 1.30[a] | | |
| 17' | 22.7 $CH_2$ | 1.29[a] | $H_3$-18' | C16', C18' |
| 18' | 14.2 $CH_3$ | 0.90 t (7.0) | $H_2$-17' | C17', C16' |

$^1$H and $^{13}$C assignments aided by 2D NMR experiments.

[a] Overlapped with other signals

**Table S6.** $^{1}H$ and $^{13}C$ NMR data of compounds **3**, **4** and **5** in $CDCl_3$

ù

| | Compound **3**[a] | | Compound **4**[b] | | Compound **5**[b] | |
|---|---|---|---|---|---|---|
| Position | $\delta_C$ type | $\delta_H$ m (*J* in Hz) | $\delta_C$ type | $\delta_H$ m (*J* in Hz) | $\delta_C$ type | $\delta_H$ m (*J* in Hz) |
| NH | - | 6.04 m | - | 6.06 d (7.1) | - | 5.87 d (7.8) |
| 1 | 176.0 C | - | 176.0 C | - | 175.7 C | - |
| 2 | 51.1 CH | 4.55 m | 51.0 CH | 4.59 m | 51.2 CH | 4.60 m |
| 3 | 40.7 $CH_2$ | 1.71 m | 41.1 $CH_2$ | 1.72 m | 40.6 $CH_2$ | 1.75 m |
| | | 1.58[c] | | 1.60[c] | | 1.65[c] |
| 4 | 24.9 CH | 1.65[c] | 25.1 CH | 1.65[c]l | 25.0 CH | 1.63[c] |
| 5 | 21.9 $CH_3$ | 0.96 d (6.1) | 21.8 $CH_3$ | 0.95 d (6.0) | 21.8 $CH_3$ | 0.96 d (6.0) |
| 6 | 22.8 $CH_3$ | 0.98 d (6.1) | 22.9 $CH_3$ | 0.97 d (6.0) | 22.9 $CH_3$ | 0.98 d (6.0) |
| 1’ | 174.2 C | - | 174.1 C | - | 174.0 C | - |
| 2’ | 36.6 $CH_2$ | 2.25 t (7.0) | 36.5 $CH_2$ | 2.24 t (7.0) | 36.6 $CH_2$ | 2.27 t (7.3) |
| 3’ | 25.6 $CH_2$ | 1.61[c] | 25.8 $CH_2$ | 1.62 m | 25.7 $CH_2$ | 1.62 m |
| 4’ | 29.7 $CH_2$ | 1.27[c] | 29.4 $CH_2$ | 1.31[c] | 29.4 $CH_2$ | 1.30[c] |
| 5’ | 29.6 $CH_2$ | 1.27[c] | 29.4 $CH_2$ | 1.31[c] | 29.2 $CH_2$ | 1.30[c] |
| 6’ | 29.6$CH_2$ | 1.27[c] | 29.7$CH_2$ | 1.31[c] | 29.7$CH_2$ | 1.30[c] |
| 7’ | 29.3 $CH_2$ | 1.27[c] | 29.7 $CH_2$ | 1.31[c] | 29.9 $CH_2$ | 1.30[c] |
| 8’ | 29.3 $CH_2$ | 1.27[c] | 27.2 $CH_2$ | 2.00 m | 27.3 $CH_2$ | 2.03 m |
| 9’ | 29.3 $CH_2$ | 1.27[c] | 130.1 CH | 5.35[c] | 130.1 CH | 5.36[c]l |
| 10’ | 29.3 $CH_2$ | 1.27[c] | 129.9 CH | 5.35[c] | 130.1 CH | 5.36[c] |
| 11’ | 29.3 $CH_2$ | 1.27[c] | 27.2 $CH_2$ | 2.00 m | 27.3 $CH_2$ | 2.03 m |
| 12’ | 29.3 $CH_2$ | 1.27[c] | 29.4 $CH_2$ | 1.31[c] | 29.9 $CH_2$ | 1.30[c] |
| 13’ | 29.6 $CH_2$ | 1.27[c] | 29.3 $CH_2$ | 1.31[c] | 29.7 $CH_2$ | 1.30[c] |
| 14’ | 31.8 $CH_2$ | 1.27[c] | 31.9 $CH_2$ | 1.31[c] | 29.2 $CH_2$ | 1.30[c] |
| 15’ | 22.7 $CH_2$ | 1.28[c] | 22.7 $CH_2$ | 1.31[c] | 29.4 $CH_2$ | 1.30[c] |
| 16’ | 14.5 $CH_3$ | 0.89 t (7.1) | 14.2 $CH_3$ | 0.89 t (6.9) | 31.9 $CH_2$ | 1.30[c] |
| 17’ | | | | | 22.7 $CH_2$ | 1.30[c] |
| 18’ | | | | | 14.3 $CH_3$ | 0.90 t (7.0) |

[a] $^{1}H$ and $^{13}C$ spectra were reordered at 700 MHz and 175 MHz; [b]$^{1}H$ and $^{13}C$ spectra were reordered at 400 MHz and 100 MHz; [c] Overlapped with other signals

**Figure S3.** $^{1}$H-NMR spectrum of compound **1** ($CDCl_3$, 700MHz)

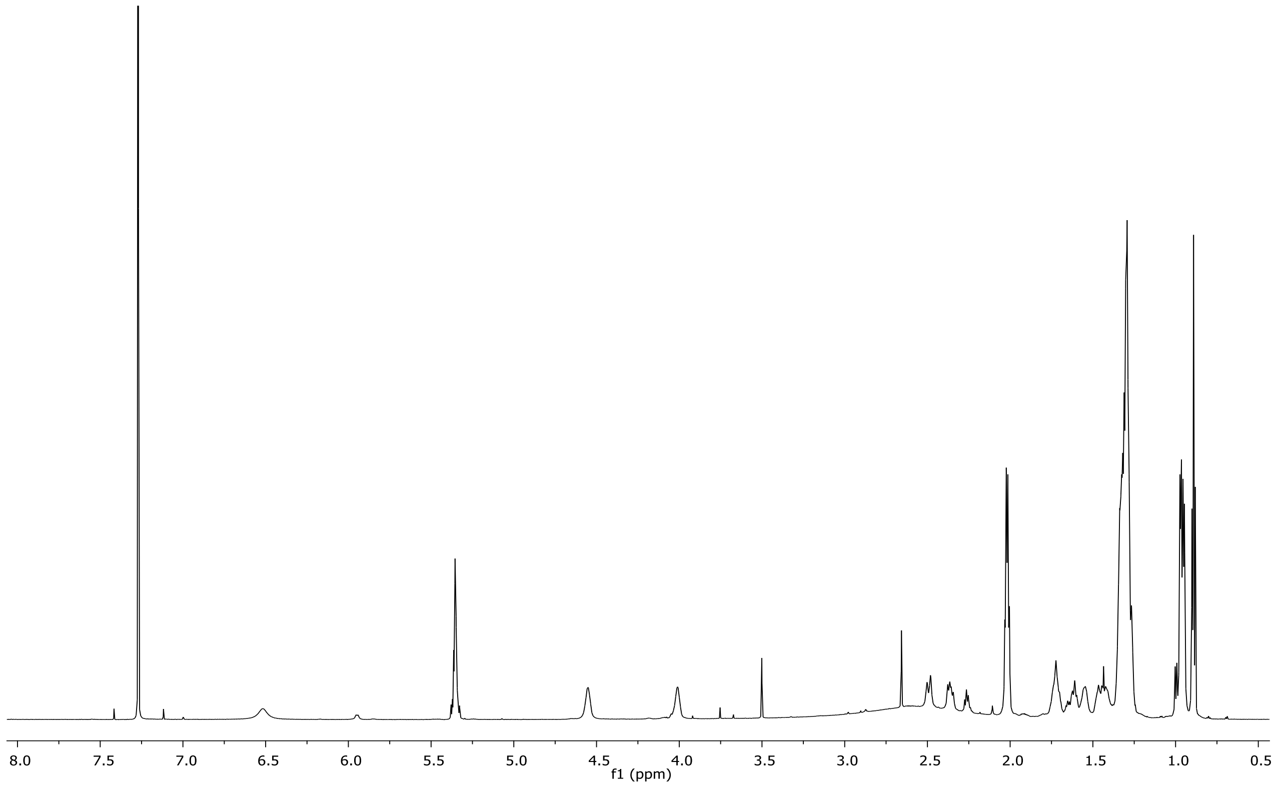


**Figure S4.** COSY spectrum of compound **1** ($CDCl_3$, 700MHz)

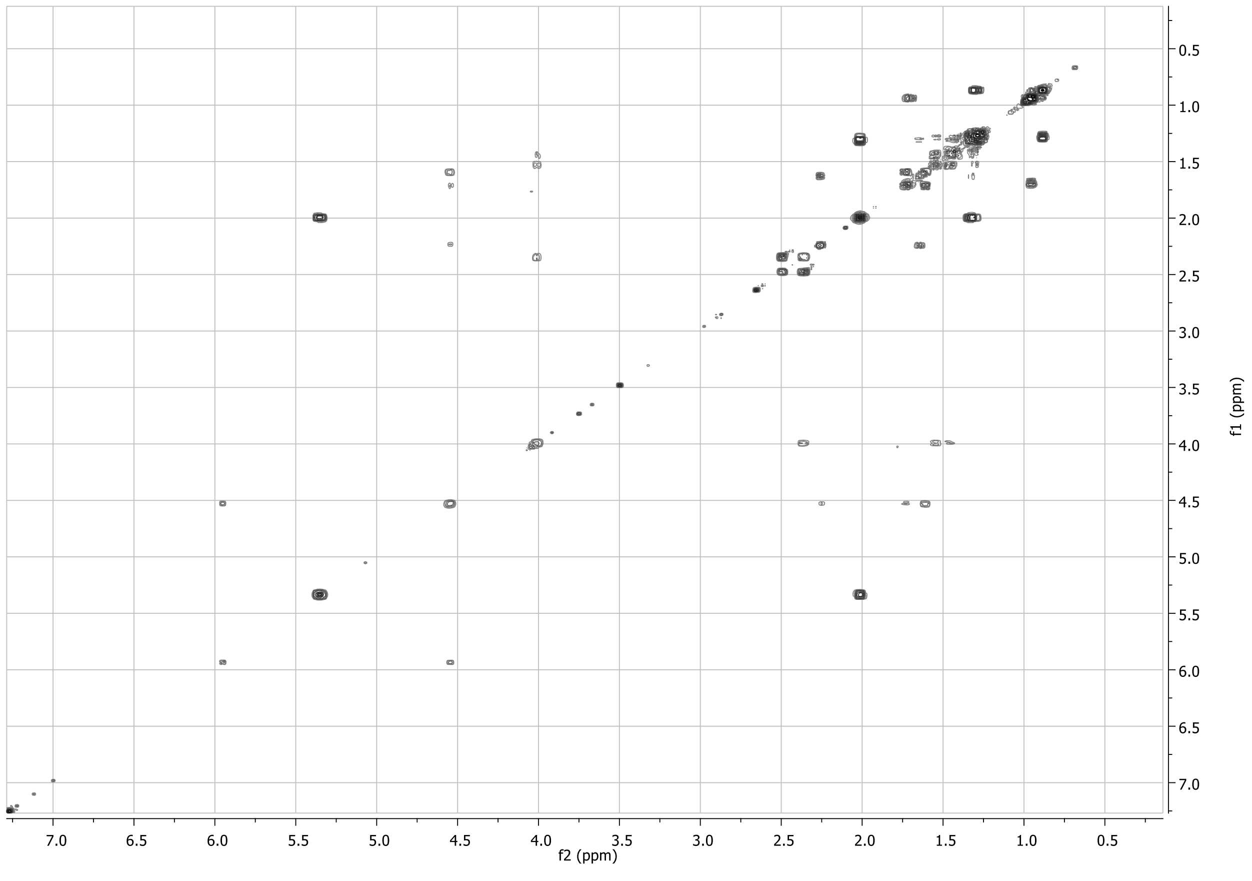

**Figure S5.** HSQC spectrum of compound **1** ($CDCl_3$, 700MHz)

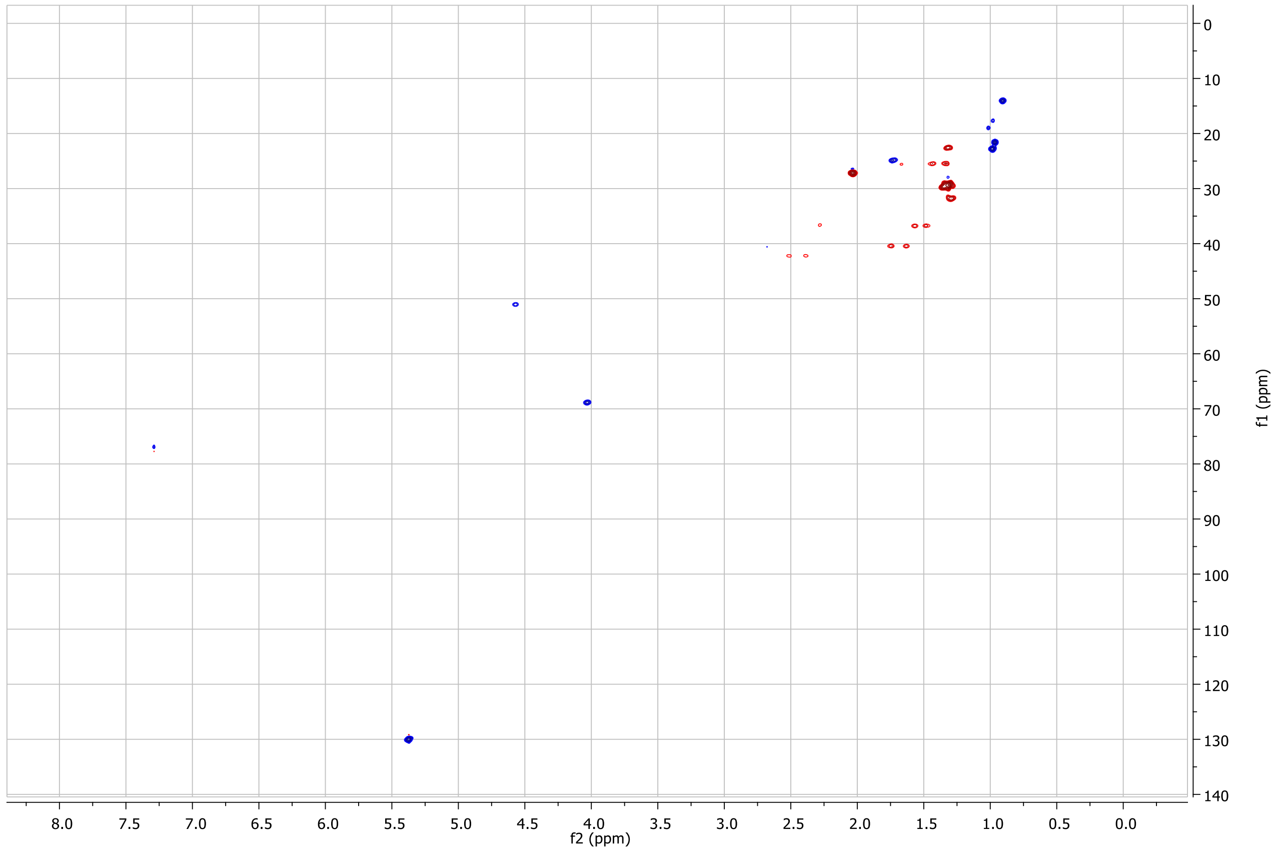


**Figure S6.** HMBC spectrum of compound **1** ($CDCl_3$, 700MHz)

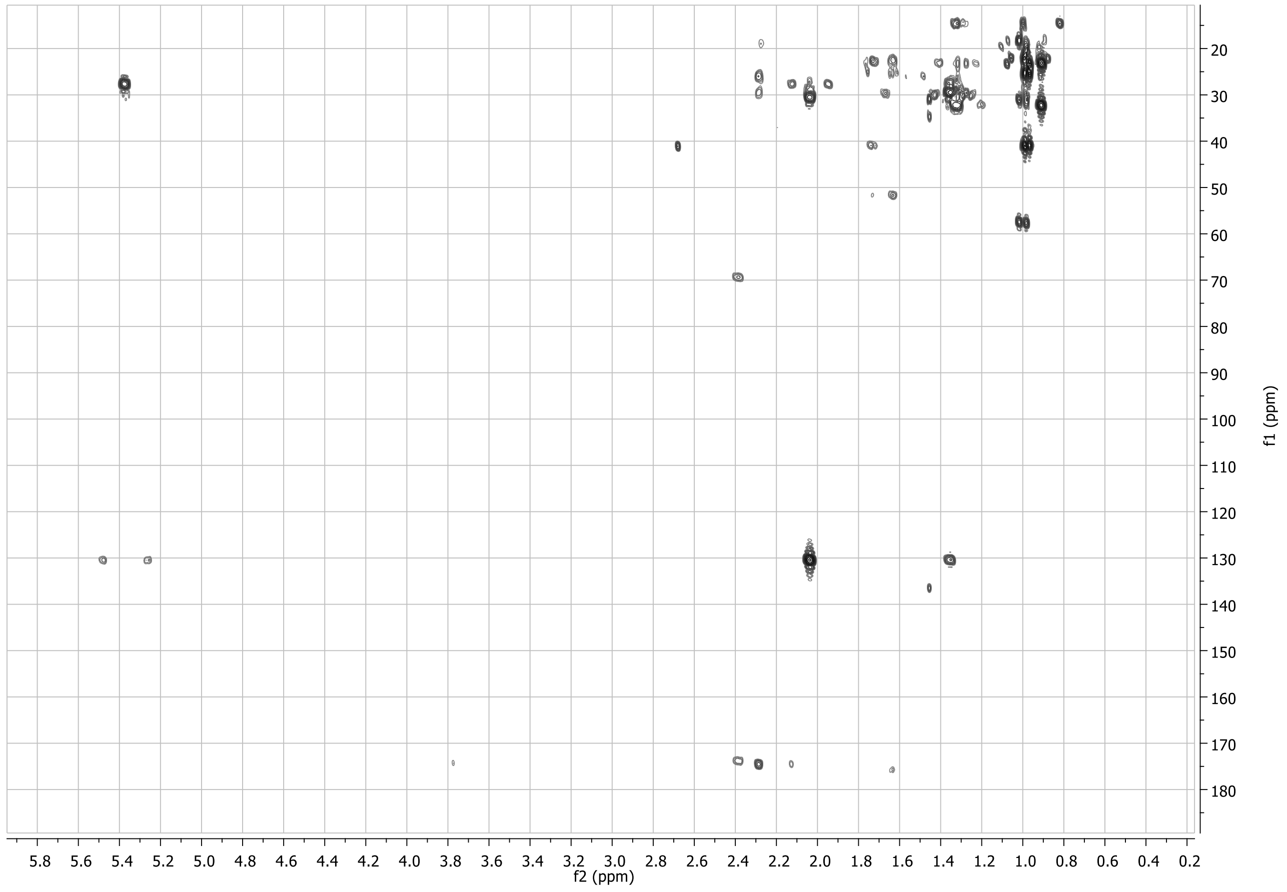

**Figure S7.** $^1$H-NMR spectrum of compound **2** ($CDCl_3$, 400MHz)

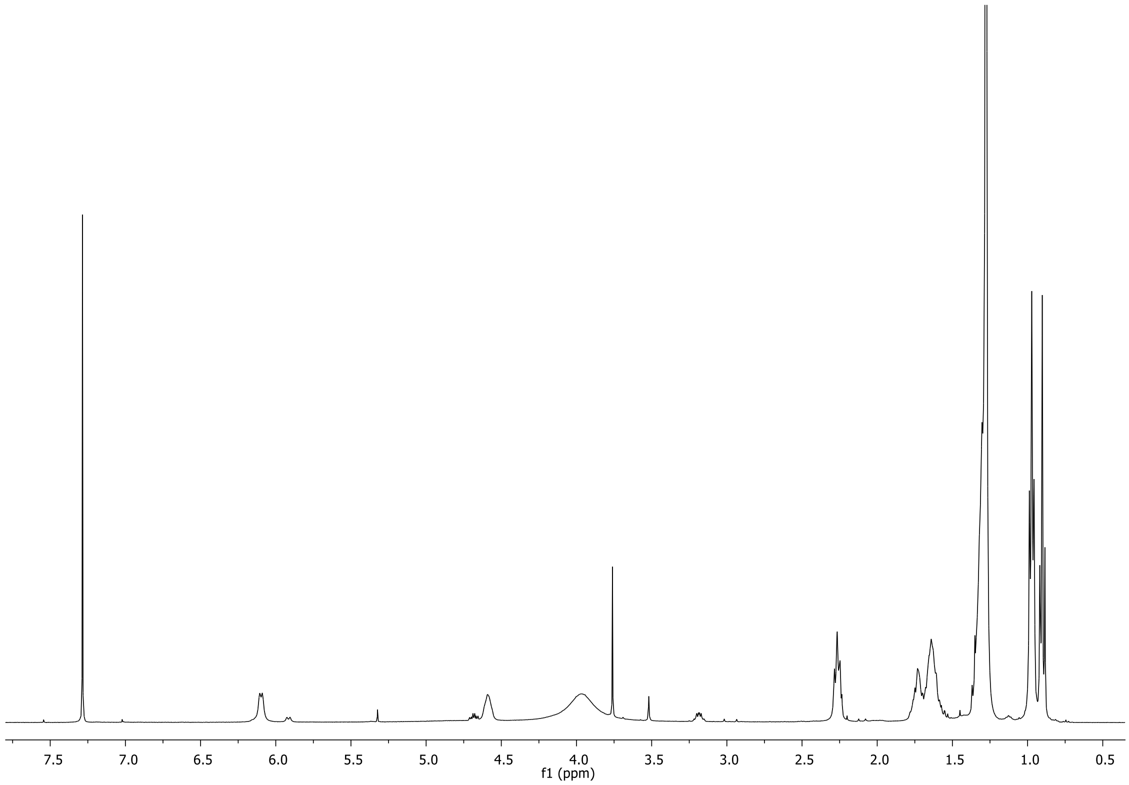


**Figure S8.** $^1$H-NMR spectrum of compound **3** ($CDCl_3$, 700MHz)

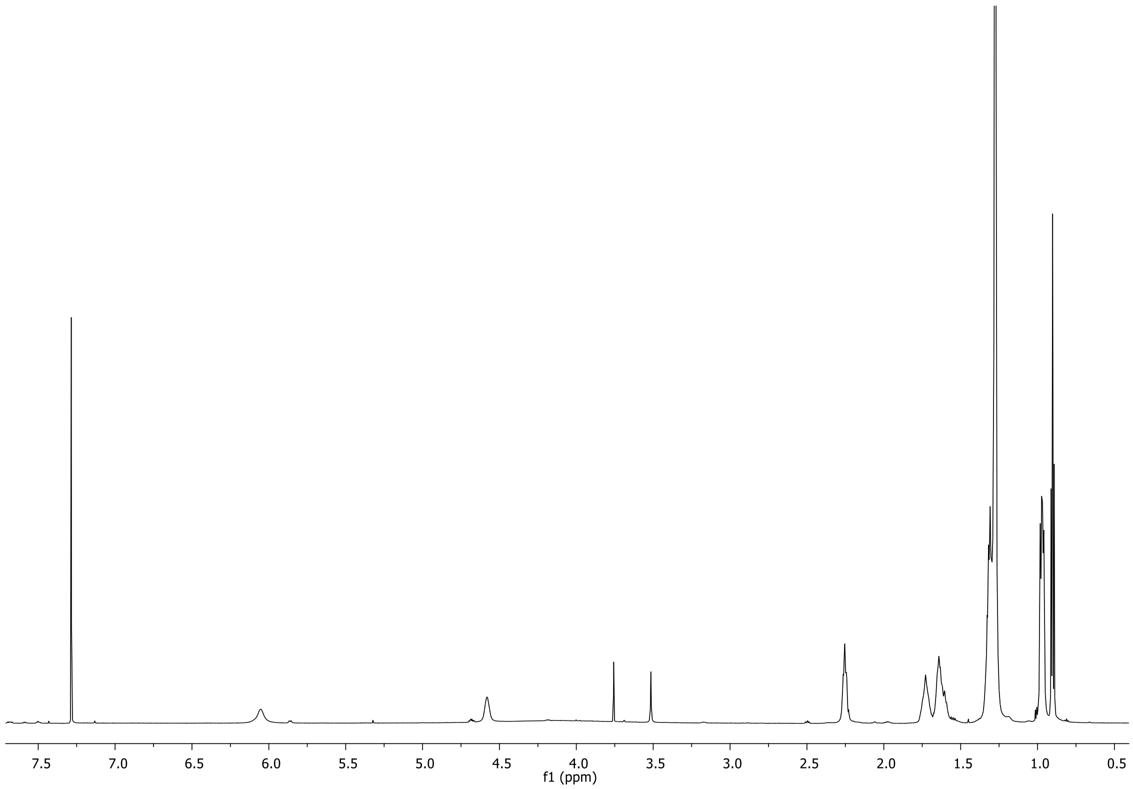

**Figure S9.** $^{13}$C NMR spectrum of compound **3** ($CDCl_3$, 175 MHz)

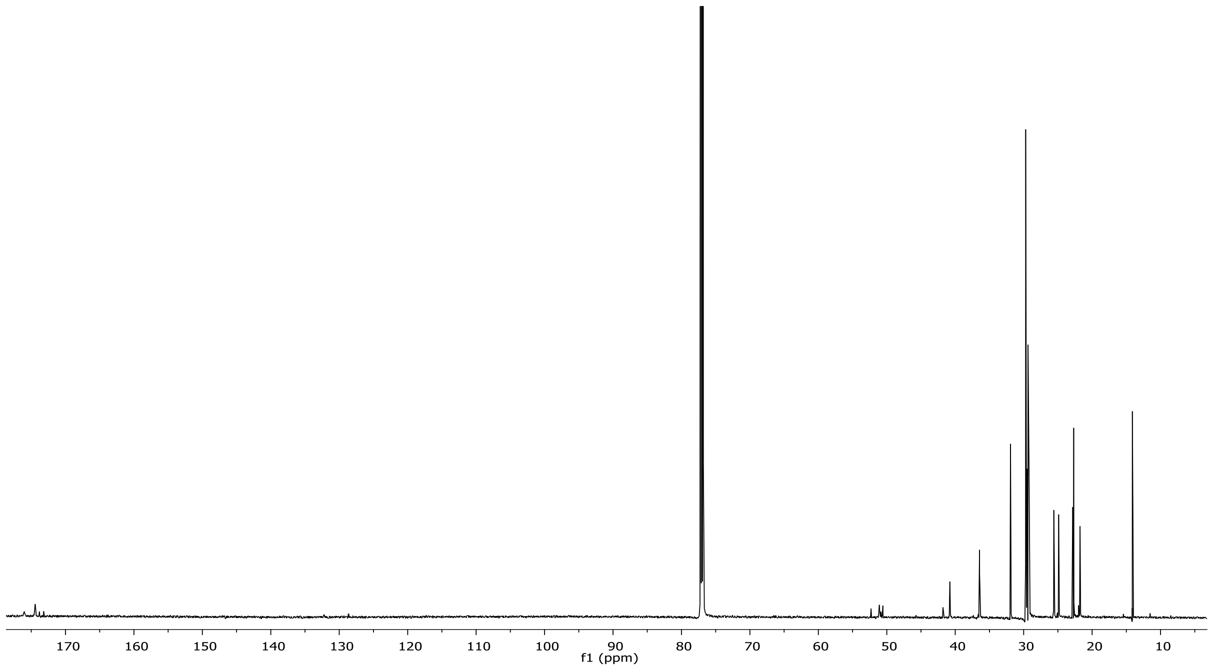


**Figure S10.** $^{1}$H-NMR spectrum of compound **4** ($CDCl_3$, 400MHz)

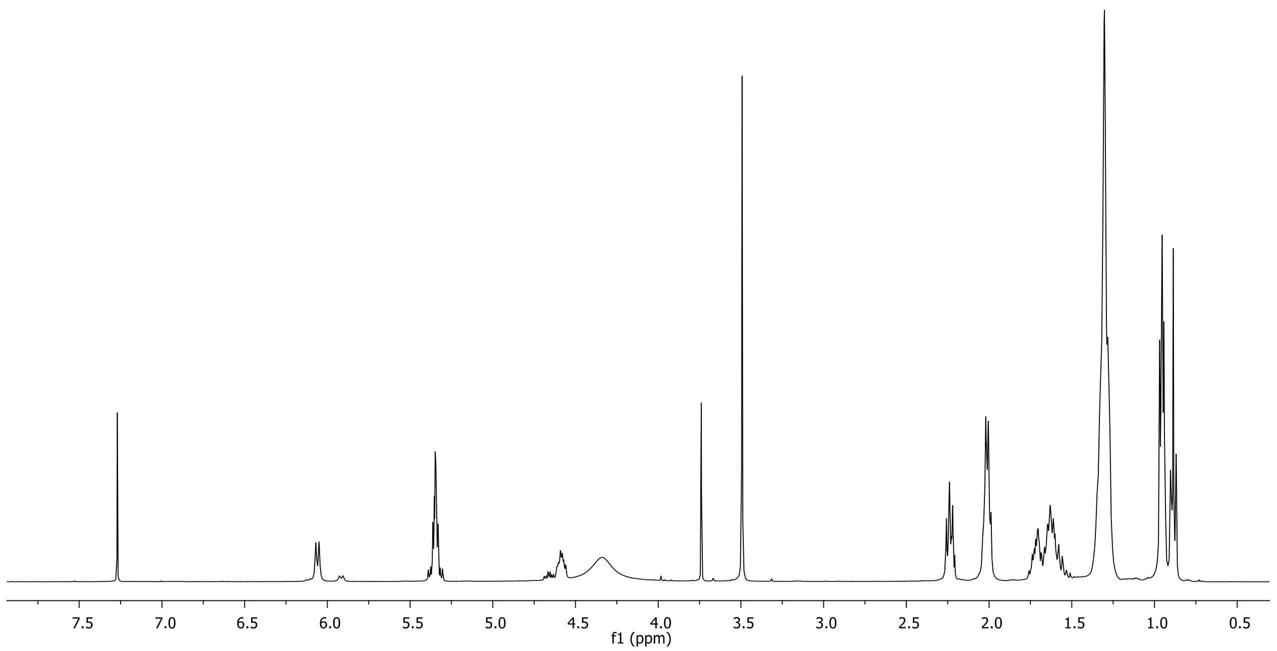

**Figure S11.** $^{13}$C NMR spectrum of compound **4** ($CDCl_3$, 100 MHz)

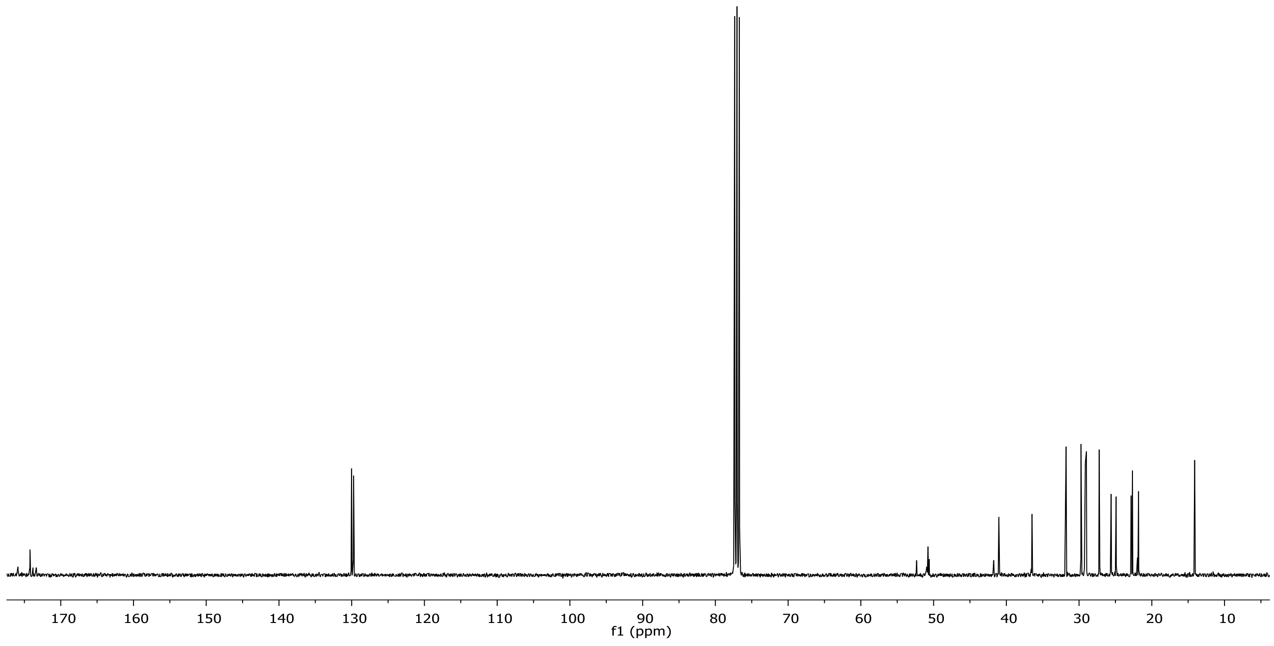


**Figure S12.** $^{1}$H-NMR spectrum of compound **5** ($CDCl_3$, 400MHz)

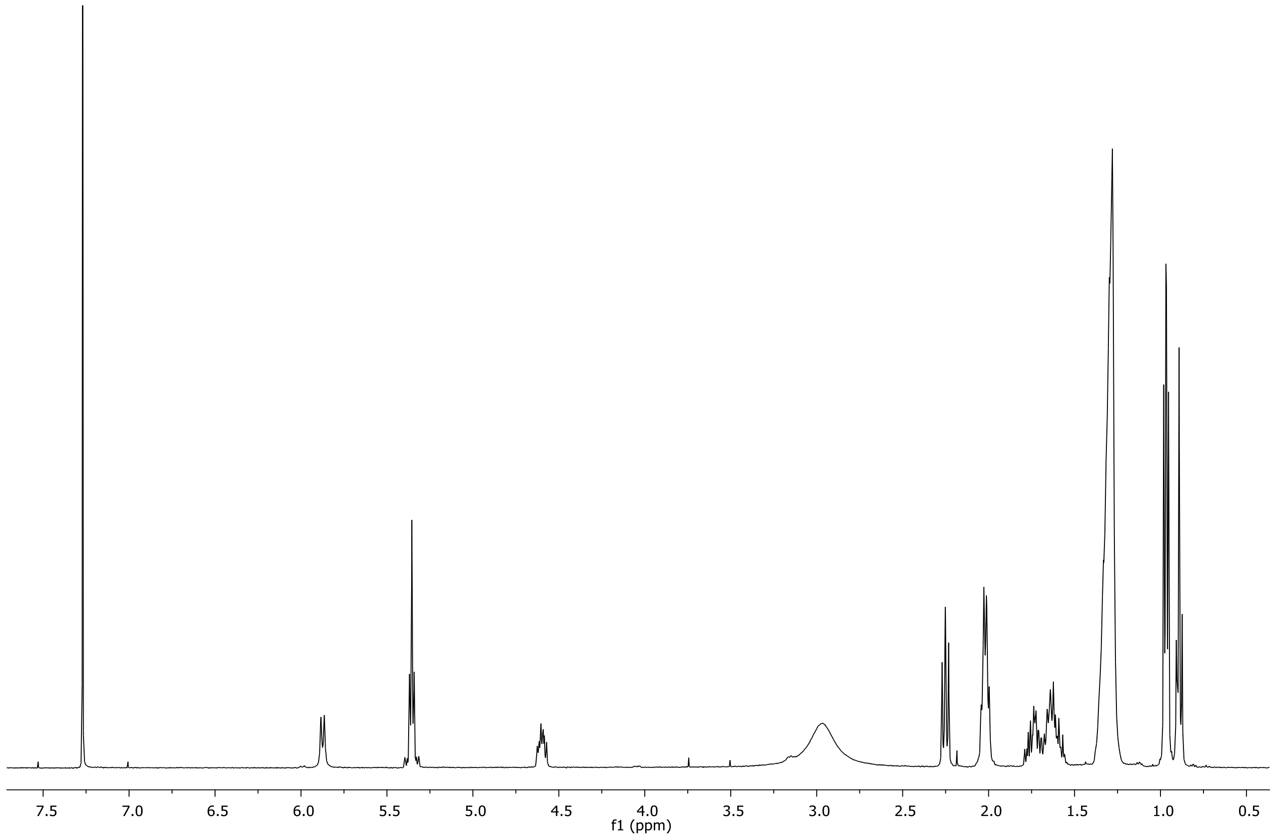

**Figure S13.** $^{13}$C NMR spectrum of compound **5** ($CDCl_3$, 100 MHz)

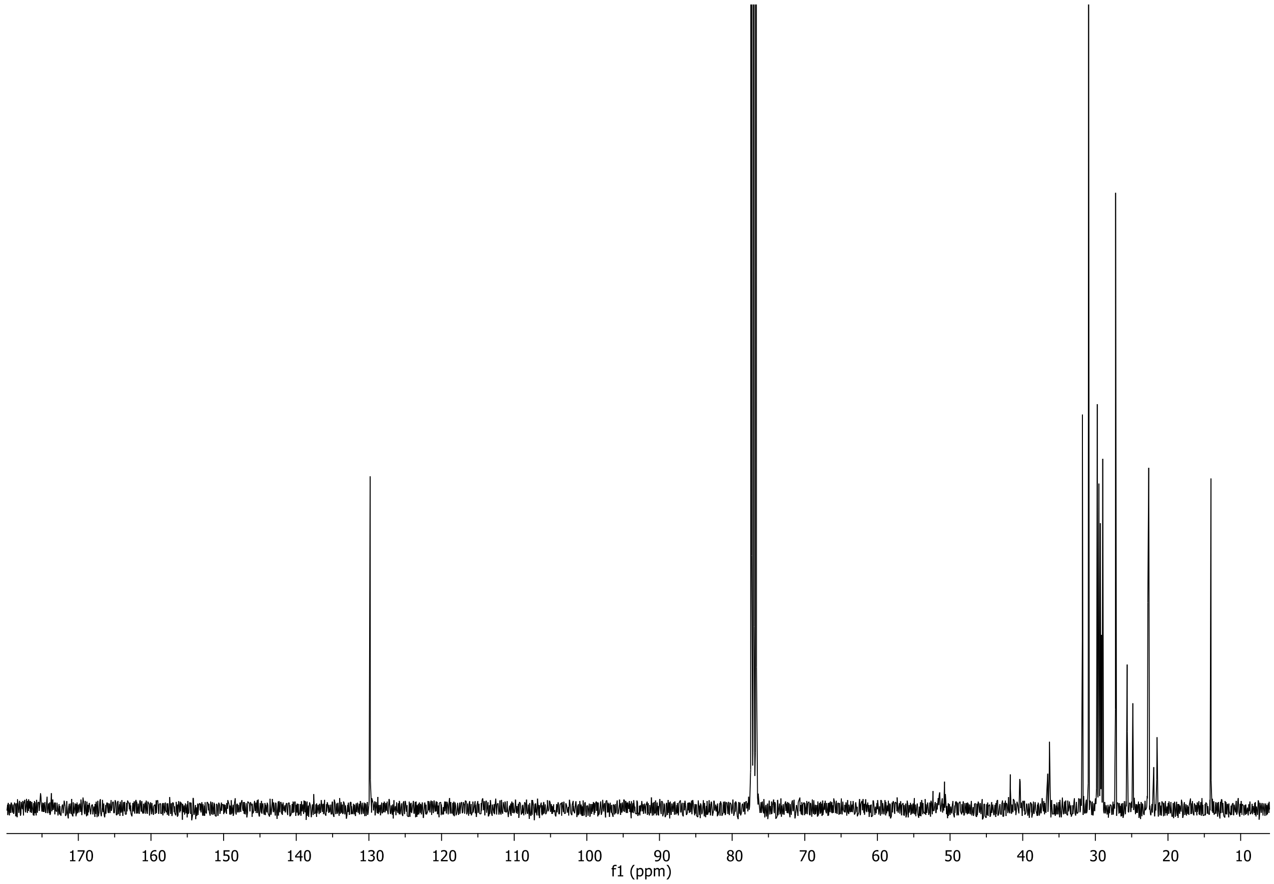


**Figure S14.** $^{1}$H-NMR spectrum of compound **6** ($CD_3OD$, 400MHz)

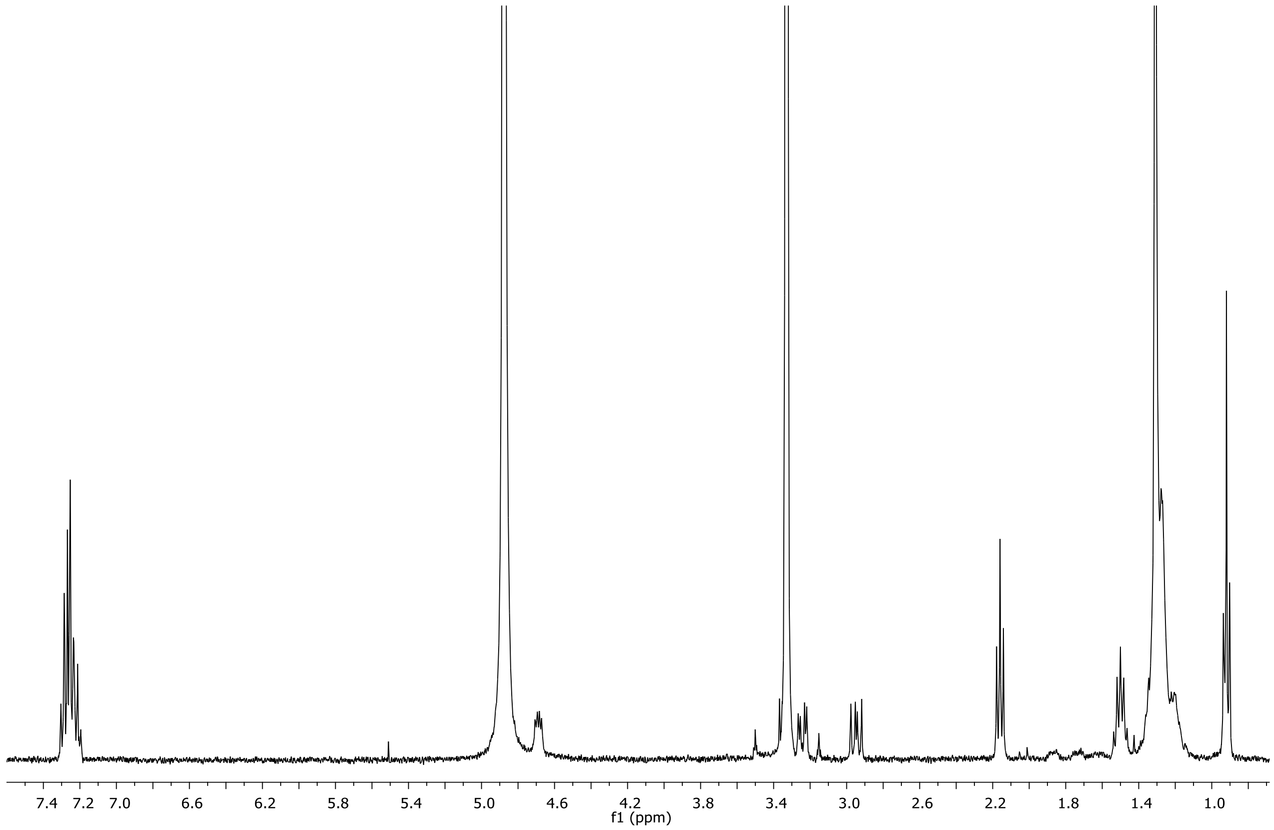

**Figure S15.** $^{1}$H-NMR spectrum of compound **7** ($CD_3OD$, 400MHz)

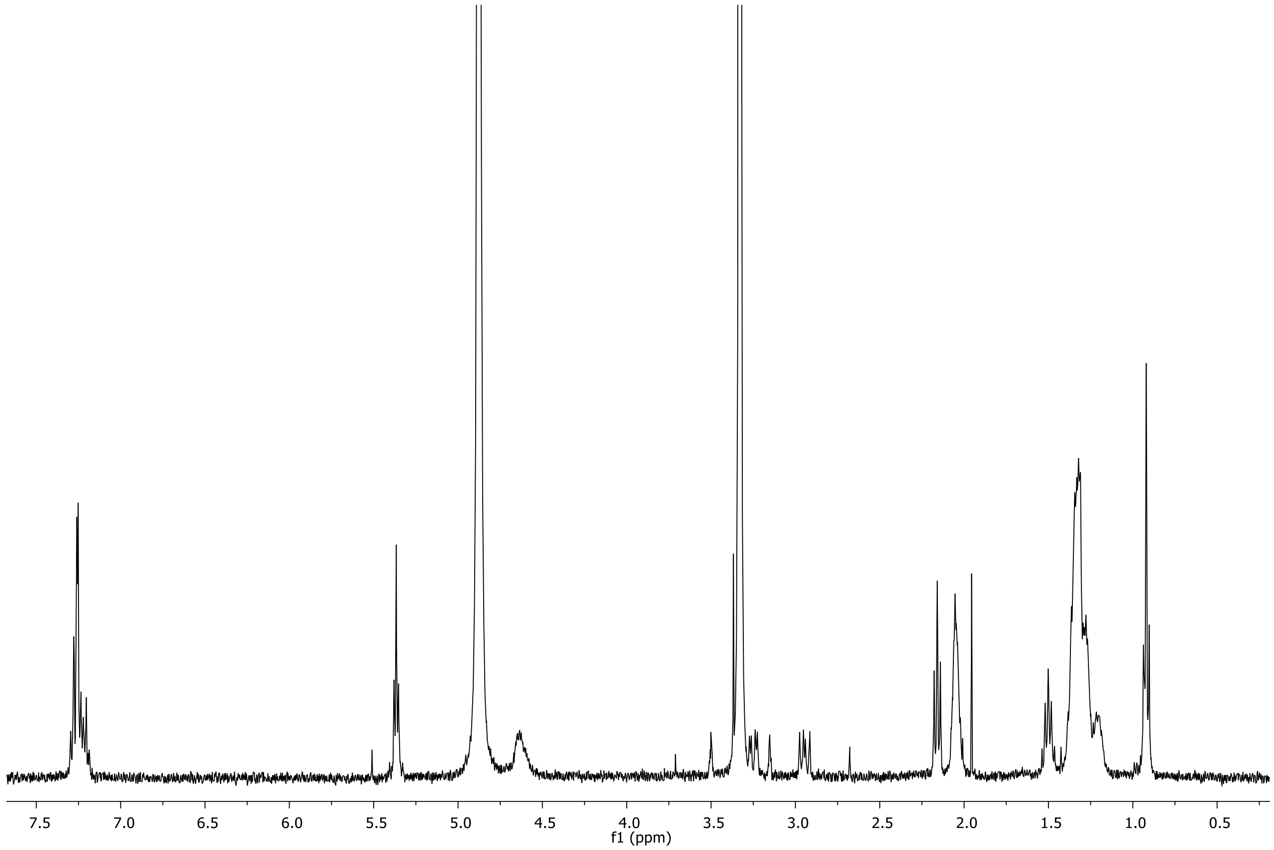


**Figure S16.** $^{1}$H-NMR spectrum of compound **8** ($CD_3OD$, 400MHz)

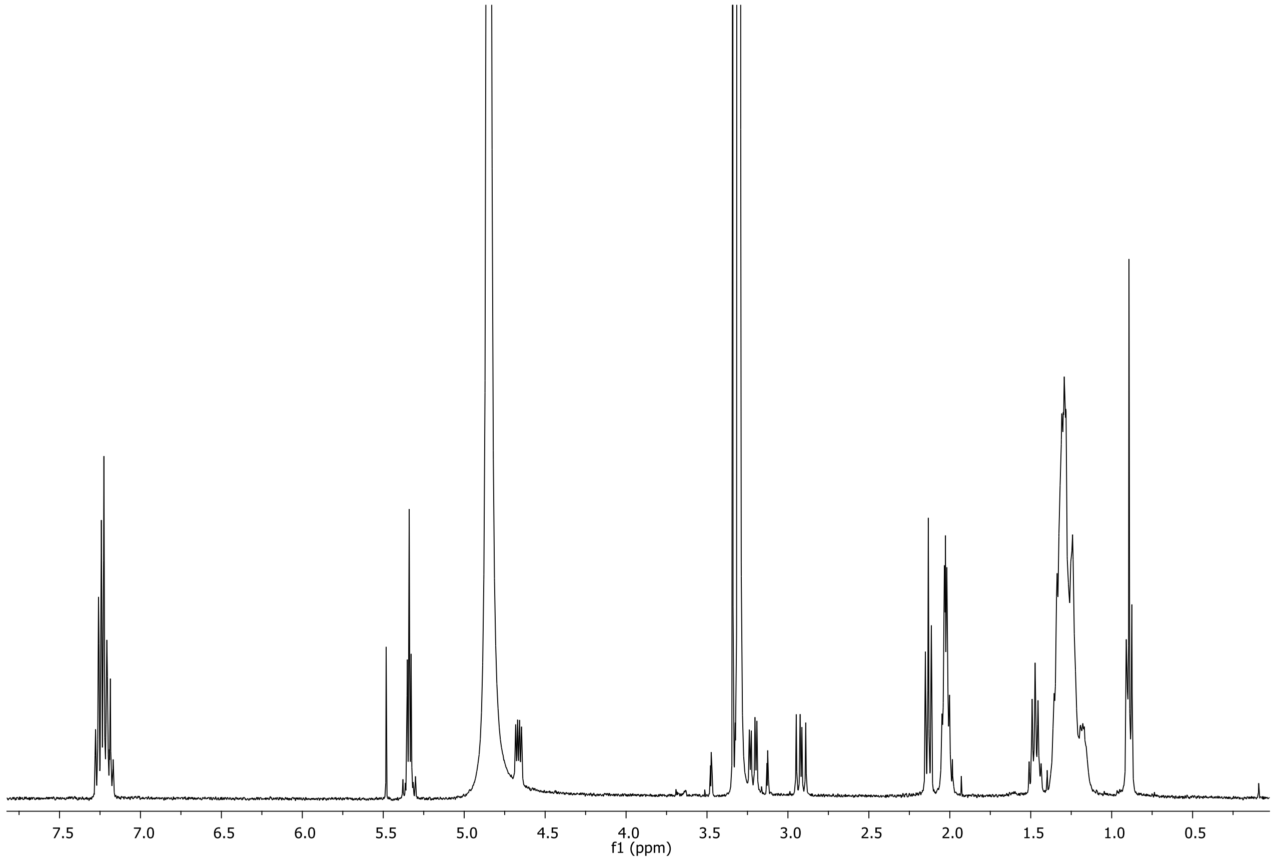

**Figure S17.** $^{13}$C NMR spectrum of compound **8** ($CD_3OD$, 100 MHz)

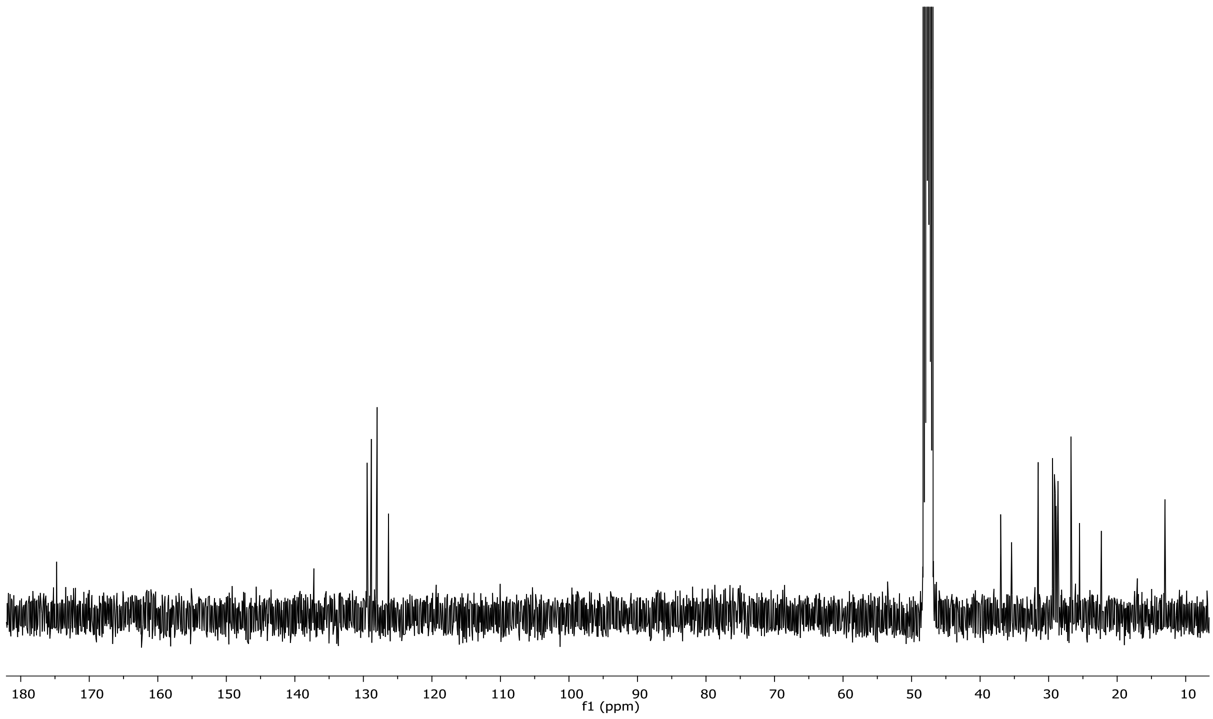


**Figure S18.** Extracted-ion chromatograms at *m/z* 384.1415 of the L- and D-FDAA derivatives of the hydrolysis product from compound **3** and of D- and L-FDAA derivatives with standard L-Leu.

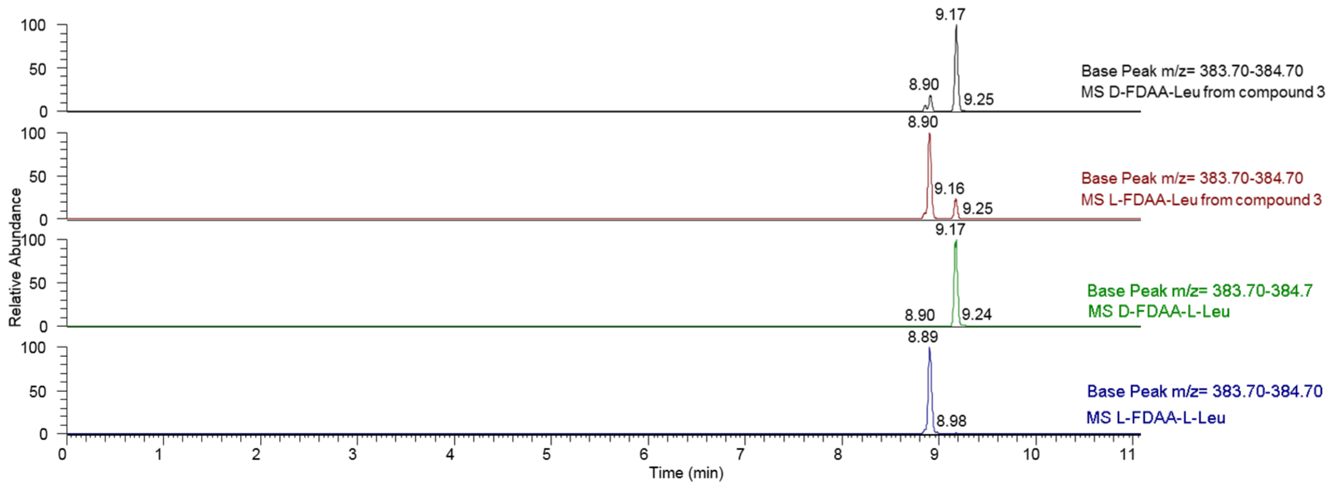

**Bibliography**


28. Pathak, K. V.; Bose, A.; Keharia, H., Characterization of novel lipopeptides produced by Bacillus tequilensis P15 using liquid chromatography coupled electron spray ionization tandem mass spectrometry (LC–ESI–MS/MS). *International Journal of Peptide Research Therapeutics* **2014,** 20, (2), 133-143.
29. Bartal, A.; Vigneshwari, A.; Bóka, B.; Vörös, M.; Takács, I.; Kredics, L.; Manczinger, L.; Varga, M.; Vágvölgyi, C.; Szekeres, A., Effects of different cultivation parameters on the production of surfactin variants by a Bacillus subtilis strain. *Molecules (Basel, Switzerland)* **2018,** 23, (10), 2675.
30. Kecskeméti, A.; Bartal, A.; Bóka, B.; Kredics, L.; Manczinger, L.; Shine, K.; Alharby, N. S.; Khaled, J. M.; Varga, M.; Vágvölgyi, C., High-frequency occurrence of surfactin monomethyl isoforms in the ferment broth of a Bacillus subtilis strain revealed by ion trap mass spectrometry. *Molecules (Basel, Switzerland)* **2018,** 23, (9), 2224.
